# Massive Gastric Hemorrhage after Indomethacin Therapy: A Rare Presentation and Critical Management in an Extremely Preterm Infant

**DOI:** 10.3390/children8070545

**Published:** 2021-06-24

**Authors:** Yen-Ju Chen, Wei-Ying Chu, Wen-Hao Yu, Chau-Jing Chen, Shu-Ti Chia, Jieh-Neng Wang, Yung-Chieh Lin, Yu-Jen Wei

**Affiliations:** 1 Department of Pediatrics, National Cheng Kung University Hospital, College of Medicine, National Cheng-Kung University, Tainan 704302, Taiwan; yensweet@gmail.com (Y.-J.C.); fieldof19@gmail.com (W.-H.Y.); jiehneng@mail.ncku.edu.tw (J.-N.W.); 2 Department of Pediatrics, Tainan Hospital, Ministry of Health and Welfare, Tainan 700007, Taiwan; raychu9629@gmail.com; 3 Department of Surgery, Tainan Sinlau Hospital, Tainan 701002, Taiwan; chenps2008@gmail.com; 4 Department of Surgery, College of Medicine, National Cheng-Kung University, Tainan 701401, Taiwan; n102677@mail.hosp.ncku.edu.tw; 5 Department of Pediatrics and Neonatology, Nagoya City University Graduate School of Medical Sciences, Nagoya 467-8601, Japan

**Keywords:** indomethacin, hemorrhage, preterm infants, surgery, gastric bleeding, coagulopathy, ductus arteriosus

## Abstract

Indomethacin has been widely used in preterm infants with hemodynamically significant patent ductus arteriosus (PDA). Gastrointestinal complications of indomethacin have been reported in 5% of treated neonates. However, massive gastric mucosa hemorrhage is a rarely reported complication. To the best of our knowledge, the infant in this report is the smallest reported in the literature to have undergone successful surgery for such a complication. A male preterm infant weighing 566 g was born at 25^2/7^ weeks of gestational age without a complicated maternal history. Soon after birth, he received nasal noninvasive respiratory support and minimal feeding. PDA was observed since the first day of life (DOL), treatments were initiated on the second DOL for the hemodynamical significance, and PDA was closed after two courses of indomethacin therapy (0.2 mg/kg). At midnight on the seventh DOL, generalized pallor, bloody gastric drainage, and a distended stomach were observed. Massive gastric bleeding was suspected. He suffered from intermittent hypotension, which was corrected with blood products and fluid resuscitation under monitoring with a radial arterial line. Gastric lavage with cooling saline was performed twice but in vain. Prior to surgical consultation, intravascular volume transfusion was given twice. An exploratory laparotomy was arranged after obtaining the parents’ consent. Blood oozing from the gastric mucosa was observed through gastrostomy and was successfully stopped via epinephrine-soaked gauze compression. After the operation, his clinical course remained uneventful, and he was discharged without neurological anomaly at two-year follow-up. Physicians need to be cautious of indomethacin’s effect on platelet dysfunction in preterm infants with multiple predisposing factors. The tendency for mucosal bleeding should be continuously monitored after indomethacin therapy.

## 1. Introduction

The ductus arteriosus is a communicating vessel between the aorta and pulmonary artery in human fetuses [1]. Soon after birth, the ductus arteriosus should be closed [2]. However, infants may suffer from continuously open or patent ductus arteriosus (PDA) due to prematurity [3]. The ductus, by seven days after birth, remains open in 2% of infants born between 30 and 37 gestational weeks and in 65% of infants born at 25 to 28 gestational weeks. The ductus is likely to close spontaneously in 73% of infants born at >28 gestational weeks [4]. With significant PDA, preterm infants may suffer hemodynamic instability and may require medication treatment or surgical interventions [5,6,7]. Several treatment strategies have been proposed. Prophylactic treatment may decrease the rates of severe intraventricular hemorrhage, the rates of pulmonary hemorrhage, and the rates of PDA ligation. However, prophylactic treatment may result in the overtreatment of PDA, and not enough strong evidence exists to support it as a routine approach. Symptomatic treatment based on clinical signs may minimize the disadvantages of late intervention but may lead to a lower closure rate and may correlate with complications of PDA [8].

Several studies have reported an increase in the risk of prematurity-related chronic lung disease after PDA ligation [9,10,11], and a watchful waiting strategy has been proposed as an alternative approach to dealing with a PDA [12]. However, pathological PDA may lead to more prematurity-related morbidities. Whether to treat PDA in preterm infants remains controversial [8].

Indomethacin has been widely used in preterm infants for prophylactic or therapeutic use against PDA [13,14]. Indomethacin causes the smooth muscle to constrict and closes the ductus arteriosus in preterm infants. Despite a number of studies demonstrating the association between indomethacin and a decreased risk of both severe intraventricular brain hemorrhage and pulmonary hemorrhage, gastrointestinal bleeding has been largely overlooked [15,16,17]. Indomethacin has been related to gastrointestinal bleeding in 5% of treated neonates [15,18] and adults [19]. In addition, ample evidence has shown the role of indomethacin in inhibiting platelet function [20]. Coagulopathy has often been addressed in extremely preterm infants and may cause worse gastrointestinal bleeding during indomethacin therapy.

This paper sought to describe an extremely preterm infant that suffered an indomethacin-related gastrointestinal emergency and to provide an overview to help clinicians become aware of its rare presentation.

## 2. Case Presentation

### 2.1. Perinatal History

The male preterm infant was born at 25^2/7^ weeks of gestational age and weighed 566 g. The infant had Apgar scores of 5 and 8 at 1 and 5 min, respectively. Except for being small for his gestational age, he had no other complicated maternal history. Soon after birth, he received nasal noninvasive respiratory support, amnio-acid infusion (3 g/kg/day), and trophic feeding (10 mL/kg/day) with breast milk as dictated by the protocol in [21]. His vital signs (heart rate at 167 beats per minute, blood pressure at 43/25 mmHg, and respiratory rate at 31 per minute) soon after birth were stable. The first arterial blood gas analysis showed a pH at 7.31, PCO_2_ at 43.5 mmHg, and HCO_3_^−^ at 21.6 mmol/L. The initial complete blood count showed white blood cell count at 7100/µL, hemoglobin count at 15.9 g/dL, and platelet count at 334,000/µL.

Hemodynamically significant PDA was noticed on the first day of life (DOL). A PDA with diameter of 0.2 cm and left to right shunting was confirmed by echocardiography, with a peak flow velocity 1.16 m/s. The infant also presented with tachycardia (up to 180 beat per minute), a widened pulse pressure (60/28 mmHg), and respiratory distress on the second DOL. The PDA was treated on the second DOL. Echocardiography at the bedside was performed following each dose of indomethacin [22,23]. Two courses of indomethacin therapy (0.2 mg/kg) were applied. The PDA was closed on the seventh DOL.

The data before therapy was initiated showed his platelet count at 331,000/µL, blood urea nitrogen levels at 34 mg/dL, and creatinine levels at 1.14 mg/dL. His urine output was 1.3 mL/kg/hour on the first DOL, and a diuresis phase started on the second DOL in the range of 3–3.5 mL/kg/hour during indomethacin treatment. Trophic feeding at 10 mL/kg/day was planned to be continued during indomethacin therapy [24] but was on hold since the second DOL.

After indomethacin therapy, his platelet count was 226,000/µL, and his hemoglobin count was 10.1 g/dL. Urine output increased to 5.1 mL/kg/hour, and his vital signs were all within the normal range (heart rate at 150–160 beats per minute and mean arterial pressure at 32–41 mmHg). The blood gas during therapy revealed mild non-anion gap metabolic acidosis.

### 2.2. Clinical Course of the Event

At midnight on the seventh DOL, generalized pallor and a distended abdomen were observed. The infant displayed apnea and desaturation episodes. Repeated echocardiography revealed that the PDA was closed. A sepsis event was suspected, and empirical antibiotic therapy was given. The ventilator support was shifted to noninvasive, intermittent positive-pressure ventilation.

**Figure 1:**
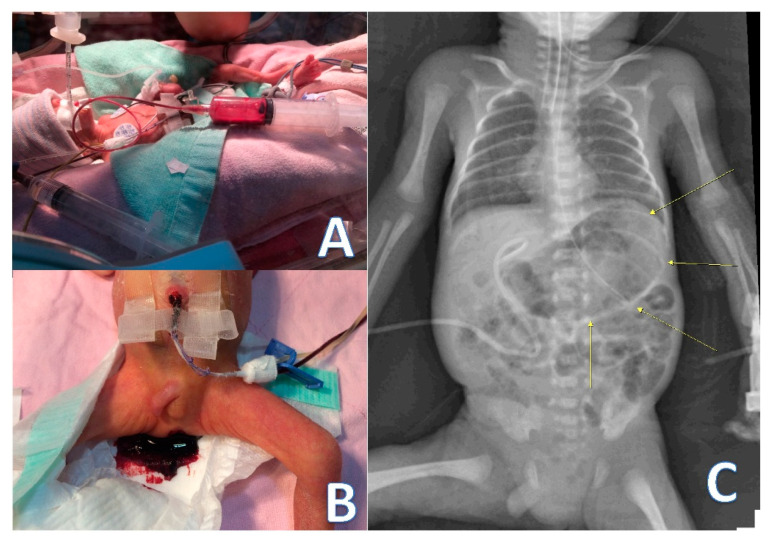
Clinical presentations and distended gastric bubble on an abdominal X-ray. (**A**) Massive blood drainage from the oral gastric tube, (**B**) dark red blood passage noted on the diaper, and (**C**) a small heart and the extensively distended gastric bubble occupied by blood (indicated by the yellow arrows).

Initially, fresh blood was found in the oral cavity and nostrils. Later, massive blood drainage from the orogastric tube was noted (Figure 1A). Pulmonary hemorrhage with swallowed blood was suspected. However, the radiography film revealed clear lung fields. The huge gastric bubble is demarcated in Figure 1C. As such, pulmonary hemorrhage was ruled out, and massive gastric bleeding was the favored diagnosis. Endotracheal intubation was performed for airway protection. No blood was aspirated from the endotracheal tube. A proton-pump inhibitor, histamine H2 receptor blocker, and vitamin K were prescribed to control upper GI bleeding.

Hypovolemic shock developed rapidly, and the infant was resuscitated with fluid administration. Up to 43.2 mL (76 mL/kg; converted by the birth weight) of fresh blood was drained from the orogastric tube. The hematocrit dropped acutely from 30.1% to 14.5% in spite of continuous blood transfusion and fluid challenge given simultaneously during this period. The red blood cell transfusions were administrated at 10–20 mL/kg each time with a slow intravenous drip according to transfusion strategies [25,26,27]. In addition, a radial arterial line was quickly set up for accurate arterial blood pressure monitoring. The targeted optimal mean arterial pressure during transfusion was between 25 and 40 mmHg.

However, the response to medical treatment was poor since packed red blood cells (60 mL/kg divided three times) and frozen fresh plasma were transfused. Pediatric surgeons were consulted for exploratory laparotomy. The medical team and the family all agreed to undertake a critical approach in this extremely preterm infant. Informed consent was given by the father after a discussion. Massive amounts of bloody stool were also noted before the surgery (Figure 1B).

### 2.3. Surgical Management and Findings

**Figure 2:**
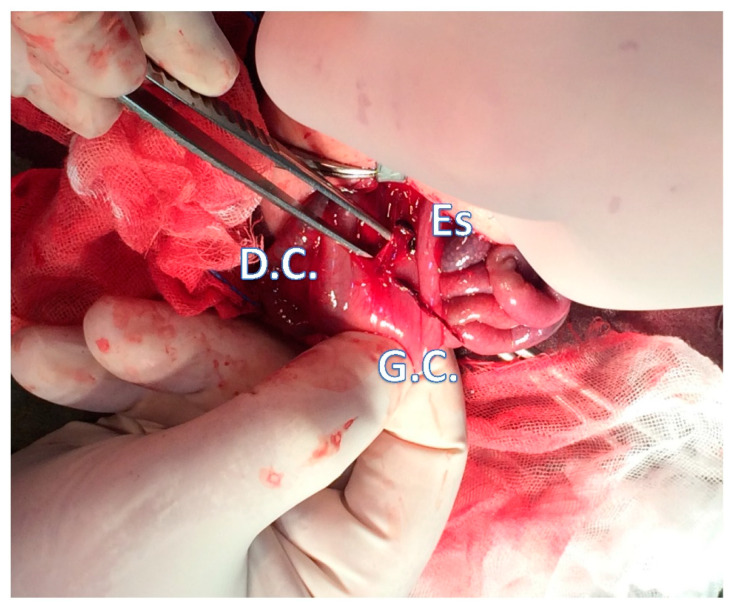
Surgical approach to the bleeding site in the lesser curvature. The bloody oozing mucosa (2 × 3 cm) was compressed with an epinephrin-soaked gauze to ensure hemostasis. Es, esophagus; G.C., greater curvature of the stomach; and D.C., duodenum c-loop.

No bleeding gastric artery was found after gastrostomy was performed on the gastric lesser curvature (Figure 2). Diffuse mucosal bleeding over the gastric fundus near the greater curvature was observed. Hemostasis was achieved via epinephrine-soaked gauze compression at the oozing site for 5 min, in which epinephrine (1:1000) was diluted to 1:100,000. After removing the gauze and confirming the lack of further bleeding, the gastric wound and the abdomen wound were closed.

During the surgery, the infant continued to receive frozen fresh plasma, platelets, and packed red blood cells. After surgery, the hematocrit recovered slightly (18.2%), and another packed red blood cell transfusion was given (20 mL/kg). The tachycardia and hypotension observed were stabilized after the surgery. His heart rate was at 159/min, and his systolic/diastolic blood pressure was 52/30 mmHg. At 6 h after the surgery, his platelet count was 86,000/µL, and his hemoglobin count was at 9.2 g/dL.

A serial cranial ultrasound revealed no intraventricular hemorrhage (IVH) after the resuscitation. However, the infant continued to pass small amounts of tarry stool until the 13th DOL.

### 2.4. Post-Operation Course

After surgery, only parenteral nutrition (110–120 kcal/kg/day with amino acids at 3.5 g/kg/day and lipids at 3 g/kg/day) was administered per our reported protocol [21]. After the tarry stool problem was resolved, the infant was extubated on the 16th DOL. We started to feed the infant 10 mL/kg/day of breast milk on the 17th DOL, and parenteral nutrition was gradually weaned when the enteral feeding amount advanced. Apnea and desaturation still occurred one to four times daily, which was diagnosed as apnea of prematurity. The septic survey showed no leukocytosis (white blood cell count at 14,900–12,000/µL), no elevated C-reactive protein (less than 0.5 mg/L), and no positive blood culture. Theophylline was given for apnea of prematurity, and hydrocortisone was administered intravenously at 0.5 mg/kg/dose every 12 h in the first 9 days and tapered to once daily for 3 days to accelerate lung maturation. The oxygen demand decreased starting on the 35th DOL. Coffee ground-like contents were occasionally aspirated from the orogastric tube, but no more fresh-blood drainage was noticed. Digestion was good, and full feeding (120 mL/kg/day) was achieved on the 85th DOL.

Ventilator support for the infant was reduced to nasal cannula flow at 0.1 L/min starting on the 100th DOL, and the infant was successfully weaned off oxygen support on the 108th DOL. The infant was discharged on the 114th DOL (postmenstrual age 41 weeks) with body weight at 2332 g, which was acceptable since he was born small for his gestational age.

### 2.5. Long-Term Follow-Up

After discharge, the patient received neurodevelopmental follow-up. The Bayley Scales of Infant Development Score, the third edition, for 24-month corrected age was normal in the cognitive, language, and motor domains. Gross motor function was normal without cerebral palsy. However, the anthropometry values were in the bottom third percentile for body weight, body height, and head circumference.

## 3. Discussion

In this report, we present an extremely preterm infant who suffered from massive gastric bleeding after indomethacin therapy for PDA. Successful resuscitation included blood-pressure-targeted fluid transfusion, an emergent surgical intervention in the form of gastrostomy, and local mucosa compression with an epinephrine-soaked gauze. Blood pressure was well corrected to within a targeted range, and no intraventricular hemorrhage occurred. No long-term neurological sequalae were found at 24-month corrected age. To the best of our knowledge, this infant may be the smallest recorded in the literature to have undergone gastrostomy and to have survived.

We propose a multi-factorial explanation for the rare hemorrhagic presentation of this patient. The relevant antecedents may be the mucosa injury from repeated hypoxia with gastrointestinal hypoperfusion due to PDA shunting (Section 3.1), extreme prematurity-related coagulopathy, the antiplatelet effect of indomethacin, and the infant’s small size for his gestational age (Section 3.2).

Due to the rare complication presented in this report, the dual effect of indomethacin on preterm infants should be further discussed and reviewed due to its popular utilization for closing the PDA (Section 3.3), and advanced management of PDA under the contraindication of indomethacin or severe bleeding should be considered (Section 3.4).

### 3.1. Gastrointestinal Mucosal Injury in Preterm Infants

The digestive system is especially sensitive to hypoxia and reduction in visceral blood flow. Experimental studies have shown that exposure of the gastric mucosa to potentially noxious factors results in little or no damage as long as adequate blood flow is maintained [28]. On the other hand, gastric injury is associated with a reduction in mucosal blood flow [29], whereas the protection and healing of gastric mucosa is associated with an increase in gastric blood flow [30,31,32]. A similar effect of blood flow organ integrity was observed in other organs of the gut, such as the duodenum [33,34], large bowel [35,36], pancreas [37,38], and liver [39]. Additionally, in necrotizing enterocolitis, a devastating intestinal disease affecting premature infants, the underlying mechanisms of this disease involve a combination of reduction in local blood flow with infection of the intestines [40].

After birth, extremely preterm infants may suffer simultaneously iatrogenic or hemorrhagic anemia, hypoxia from immature respiratory system, and hemodynamic significant PDA, which had a shunting effect leading to hypoperfusion of the gastrointestinal system. Those morbidities may synergistically contribute to gastrointestinal injury in preterm infants [41]. Multidisciplinary and aggressive prevention may reduce preceding factors of mucosal injury. Recently, delayed cord clamping in term and preterm infants has been a simple, safe, and effective delivery procedure leading to the prevention of anemia and to improvements in infant condition [42]. In the unit of this reported infant, umbilical cord milking after birth is a routine for peri-viable infants [43] and may have a similar effect to delayed cord clamping [44].

### 3.2. Coagulation Issue in Extremely Preterm Infants

Thrombocytopenia is frequently observed in preterm infants compared to term infants [45,46]. Thrombocytopenia has been related to perinatal morbidities, including being small for gestational age, having maternal hypertension, experiencing disseminated intravascular coagulation, having a bacterial infection, having a fungal infection, and experiencing necrotizing enterocolitis [47].

The coagulation function in preterm infants may be exacerbated after indomethacin therapy. Impaired platelet aggregation was reported in infants treated with indomethacin in the 1970s [20]; the duration of impairment after indomethacin treatment is around 9–10 days.

In addition, healthy, preterm infants with very low birth weight have reported prolonged prothrombin time and activated partial thromboplastin time with slight hypocoagulability compared to term infants [48]. However, the prophylactic use of fresh frozen plasma in extremely preterm infants has declined and is not recommended [25,49].

### 3.3. Pros and Cons of Indomethacin in Hemorrhagic Diseases of Preterm Infants

Indomethacin has been assessed in many studies [16,50,51,52,53,54] and has been known to be effective for the closure of the patent ductus arteriosus since the early 1980s [55]. Other than its role in platelet dysfunction [50,56], the use of indomethacin for the prevention of severe neonatal intraventricular hemorrhage has been investigated by Ment et al. [51] and other research teams [57,58,59] since the late 1980s. Afterwards, considerable research provided convincing evidence in favor of prophylactic indomethacin use in extremely preterm infants to decrease mortality and pulmonary hemorrhage [60,61,62].

A platelet count of <50,000/µL was the only screening test prescribed before the use of prophylactic indomethacin, [63], and prolonged prothrombin time and activated partial thromboplastin time were seldom mentioned. The research seems to have largely overlooked this risk associated with severe bleeding beyond the ventricles and lungs. Gastrointestinal bleeding and ulceration are well known to be associated with non-steroid anti-inflammation drugs [19], including indomethacin and ibuprofen. Gastrointestinal complications have been reported in 5% of treated neonates [15,18]. However, massive gastric mucosa hemorrhage, as in this case, is a rarely reported complication and requires further surgery. In the literature, Ryan et al. reported on six extremely low-birth-weight infants with large subcapsular hemorrhage of the liver and proposed the administration of indomethacin as a relevant antecedent event [64].

Some scholars have claimed that the tailor-made management of PDA in the early life of extremely preterm infants decreases the total doses of indomethacin required [8,23,65]. Su et al. undertook a doppler echocardiographic assessment of the PDA shunt flow pattern as a guide to decide whether to treat the PDA [22,23]. A pulsatile or growing PDA flow pattern represents a falling pulmonary resistance combined with a not completely restricted PDA, which contributes to clinical significance in hemodynamics. Treatment guided by serial changes in PDA flow pattern significantly reduces the total doses of indomethacin treatment [23]. A reduced total dose and a reduced rate of complications for indomethacin have been reported without a decreased rate of closure of the PDA. The study unit’s policy of PDA management was described in our previous report [43].

In summary, physicians need to be cautious of indomethacin’s anti-platelet effects when considering its benefits in the prevention of severe intraventricular hemorrhage and pulmonary hemorrhage.

### 3.4. Management of Hemodynamic Significant PDA in Preterm Infants with Coagulopathy or Active Bleeding

The management of hemodynamically significant PDA in preterm infants with coagulopathy or active bleeding may be difficult. Nonsteroidal anti-inflammatory drugs, including indomethacin or ibuprofen, may worsen the tendency to bleed, and surgeons may be concerned about bleeding during the operation. Fortunately, this patient’s ductus arteriosus did not open after the abdominal surgery.

Paracetamol has been reported to be as effective as ibuprofen or indomethacin, and paracetamol may pose a lower risk of gastrointestinal bleeding than ibuprofen or indomethacin [66,67]. However, paracetamol is emerging in research on treating hemodynamically significant PDA preterm infants [68], and future studies will have to take neurodevelopmental follow-up into account [69,70].

In preterm infants with pulmonary hemorrhage and hemodynamically significant PDA who are not able to undergo surgical ligation, some authors have reported transcatheter occlusion as a feasible and alternative approach [71,72]. Less invasive procedures, such as transcatheter closure, may be of use to this critical population [72,73].

## 4. Conclusions

Massive gastric bleeding is a possible and rare but severe complication arising in the combination of PDA, anemia, hypoxia, hypoperfusion, extreme prematurity, being small for gestational age, and indomethacin therapy. Early surgical intervention for intractable hemorrhage may be successful without long-term neurodevelopmental sequalae.
